# Misguided and Modest: Reflections of Our Youth Voice Research

**DOI:** 10.3389/fspor.2022.824953

**Published:** 2022-07-05

**Authors:** Annette Stride, Ruth Brazier, Hayley Fitzgerald

**Affiliations:** Carnegie School of Sport, Leeds Beckett University, Leeds, United Kingdom

**Keywords:** youth voice, young people, participatory methods, power, change

## Abstract

In recent years, Physical Education (PE) has seen a growth in the commitment to youth voice research. This approach foregrounds the practice of researching with young people, rather than conducting research on or about them. Whilst we are cognisant of the many possibilities youth voice research offers, we are also concerned that there is a tendency to overlook the challenges of supporting youth voice activities. This paper draws on our collective reflections to bring to the fore some of the complexities we have encountered when attempting to engage in school-based youth voice research. We explore the following questions: How can youth voice research engage with different young people to capture a diversity of voices? What are the challenges of undertaking youth voice research? What are the possibilities of change through youth voice research? We consider these questions by drawing upon four principles of student voice work including communication as dialogue, participation and democratic inclusivity, unequal power relations, and change and transformation. We use these principles to critique our own research and, in doing so, draw on entries from our research diaries. The paper questions whether young people need help to share their insights and experiences about PE, or whether it is us - researchers, teachers and schools - who need help to more readily recognize and be attentive to young people's voices. We also point to the importance of recognizing modest change through youth voice research and the need to secure adult allies to support activities and potential outcomes. Engaging in youth voice research is an immersive and messy encounter that involves navigating a journey that is anything but straightforward. Even though this is the case, our moral and ethical compass continues to point us in this direction and we remain firm advocates of youth voice work. This paper offers a starting point for others to begin to grapple with the pitfalls and possibilities when supporting youth voice research.

## Introduction

*Facilitating the participation of agentic children and asking that they share their voice does not always lead to children's truths* (Clark and Richards, 2017, p. 142)

In recent years, Physical Education (PE) research has increasingly advocated working *with* young people rather than viewing them as objects to be studied (see for example O'Sullivan and MacPhail, 2010; Azzarito and Kirk, 2013). In part, this outlook promotes youth voice and recognizes the importance of enabling marginalized and silenced voices to be heard. Indeed, this Research Topic is testament to the growing appetite scholars have to foreground the voices of young people within PE and sports research. Youth voice research typically refers to the active participation of young people in the research process (Hadfield and Haw, 2001). This can cover a whole gamut of possibilities: the use of participatory methods in generating data; including young people in decision making around the research focus and ways to collect data; and their involvement in analysis and dissemination activities (Woodgate et al., 2020). It is an approach that positions them as agents and curators of knowledge, privileges their experiences, and offers opportunities for empowerment (Cook-Sather, 2007; Gallacher and Gallagher, 2008). We have utilized the term youth voice in our research projects where any of these possibilities have been attempted. Advocates of youth voice research extoll the virtues of this kind of work (Holland et al., 2010; Jones and Bubb, 2020), and whilst we are aware of the opportunities that approaching research in this way can offer, we are also concerned that there is a tendency to overlook the challenges of attempting to promote youth voice. In relation to research, as we have highlighted elsewhere, this is evident when publications present a rather sanitized account of the research journey (Fitzgerald et al., 2020). We concur with Finneran et al. (2021) and others (McNamara, 2011; Fox, 2013; Bradbury-Jones and Taylor, 2015), that there is a need to interrogate more fully the claims researchers make about promoting youth voice within research. As James (2007, p. 270) argues, it is only through reflecting upon “the complexities of the issues that frame what children say, rather than offering the simple message that recording and reporting their voices is sufficient, [that] it may be that children's voices will be more willingly listened to and their perspectives more readily understood”. We feel a duty to be more open about our research endeavors including the difficulties and dilemmas experienced. We are firm believers that this openness can be reassuring, rather than acting as a deterrent. For example, in our discussions with early career researchers our message is clear—researching with young people can be challenging and no matter how meticulous your planning, this can go awry. When sharing the uncertainties we have experienced we have noted an almost palatable and collective sigh of relief from others, one that is later reinforced verbally with grateful feedback for our willingness to be open about our vulnerabilities. Our idea for this paper emerged from these kinds of discussions, as well as those that we have had with each other. We believe that this kind of transparency is critical for anyone committed to the youth voice project.

Whilst we acknowledge that PE is a relative newcomer to the field of youth voice research, in comparison to other disciplines including education, youth studies and health and social care (see for example, the Special Issue of Forum in 2001 (volume 43, issue 2) and of the Educational Review in 2006 (volume 58, issue 2), like James (2007) and Clark and Richards (2017) we believe there is a healthy body of work within PE that is worth taking stock of. Of course, this is necessary if we wish to develop knowledge, not only around young people's experiences, but also advancing the theory, methodology and methods informing this kind of research. In offering our insights, we are not suggesting this is in any way an expert guide that explicitly details the how and how not to undertake this type of research. Rather, we use this paper as a platform to offer some considerations that have emerged from our discussions and address the following questions:

1. How can youth voice research engage with different young people to capture a diversity of voices?
2. What are the challenges of undertaking youth voice research?
3. What are the possibilities of change through youth voice research?

We explore these questions by drawing on Robinson and Taylor's (2007) four principles of student voice^1^ work. We use these principles to critique our own research and, in doing so, offer insights into the kinds of discussions we have had with each other and the entries we have made in our research diaries. Our research diaries have proven to be a useful resource for documenting the practicalities of our research, and recording how our decisions, actions and beliefs contribute to the research process. They have also served a cathartic function, helping us to better understand our research experiences—the frustrations and joy, highs and lows, and moments of confusion and epiphany that we have encountered. In drawing upon our own research experiences, we will provide examples from two specific research projects that Annette and Ruth have each undertaken. It should be noted that whilst these projects were entirely separate from each other, they have been the focus for many of our research discussions. These conversations have encouraged us to reflect on our motives and aspirations behind researching with young people, and the realities of these projects. For this paper, we have not utilized a formal analytical approach to foreground particular research discussions or diary extracts. Rather, we have chosen those that most resonate with the research questions guiding this paper. The two research projects we draw upon are outlined next. This will help to contextualize our discussions and diary extracts, before we move on to explore the notion of youth voice research within PE. To draw the paper to a close we offer a number of concluding remarks that have emerged from our student voice work.

## Setting The Scene: Our Student Voice Research

We have each engaged in different student voice research projects over the last 15 years. In this paper we focus on two specific projects undertaken by Annette and Ruth. Annette focuses on a student voice project that explored South Asian, Muslim girls' experiences of PE and physical activity. Annette spent 2 years in a secondary school, based in the north of England. During this time she engaged in over 60 h of observations of PE lessons, worked with 23 girls, aged between 14 and 16, in four focus groups over a 4 week period, and followed this with in-depth interviews with 14 girls. At the focus group stage Annette worked with the girls in a number of participatory ways, encouraging them to create a series of research artifacts. These included posters depicting their lives, PE boxes reflecting their likes and dislikes in PE, maps that demonstrated their movements away from school, and a series of workbooks and task sheets. Annette used the various data sources to craft a series of critical, creative non-fictional narratives^2^ for each girl to ensure the voices of the girls were centralized (Stride, 2014).

Ruth draws on a student voice project exploring how the intersections of disability, ethnicity and gender influence experiences of PE. The girls involved in the research were all aged between 11 and 12 years, all classed as having a Special Educational Need or Disability (SEND), and varied in terms of their ethnicity (for example, South Asian, White British and White Slovakian). The data collection took place over 2 years in a mainstream secondary school that had a special unit for students with complex or severe SEND. Ruth conducted over 200 h of observations and engaged with 13 girls on a project they titled “PE and Me”. Through a series of eight focus group sessions girls were encouraged to share their experiences, thoughts and feelings about PE. In addition to verbal discussions each girl was given a personal research book that they could use to share information through non-verbal means (for example, mind maps, collages, drawings, and written stories). Like Annette, to foreground the girls' voices, Ruth crafted a series of critical, creative non-fictional narratives.

## Youth Voice and Research

Contemporary society recognizes knowledge production as contextual, partial, shifting and imbued with relations of power. Relatedly, there has been a change in the way young people are positioned, acknowledging them as competent social actors and experts in their own daily lived social realities. This contrasts to previous understandings of young people as “cultural dopes” (Prout and James, 1997), incapable of observing and reflecting upon their lives and the world(s) they occupy (Van Blerk and Kesby, 2009; Clark and Moss, 2011). This shift acknowledges the need to listen to and value young people's voices to gain a better understanding of their experiences. Such thinking has permeated the human rights movement (for example, the United Nations Convention on the Rights of the Child), filtering into legislation [for example, the Education Act (2002)^3^ and the Children Act (2004)^4^], and policy [for example, Every Child Matters (HM Government, 2004)] within the UK. Research too has been influenced, with an ever growing number of projects engaging young people and involving them in decision-making (James et al., 1998; Save the Children, 2000; Robinson and Taylor, 2013; Charteris and Smardon, 2019; Jones and Bubb, 2020). Such approaches need a reconsideration of the ways in which research is undertaken. As Greig et al. (2013) note, previous assumptions regarding young people's inability to contribute meaningfully to research have influenced not only the kinds of research questions that have been posed but have also delayed the development of more appropriate methods. Clark and Moss (2011) concur, citing a need to rethink more traditional approaches when researching young people's experiences. In this regard, within PE research there has been a move to embrace more innovative and creative methods that shift the emphasis to researching *with* young people as opposed to conducting research *on* them (O'Sullivan and MacPhail, 2010; Azzarito and Kirk, 2013). Whilst it is beyond the scope of this paper to review this body of work, we would draw the reader's attention to the variety of methods employed including, photo elicitation (Azzarito and Hill, 2013; Stride, 2014), drawings (Fitzgerald, 2012), media exploration (Hamzeh and Oliver, 2012; Enright and O'Sullivan, 2013) scrap books and journals (Hamzeh, 2011) and vlogs (Sharpe et al.'s, 2021). Moreover, we also pay homage to scholars who are experimenting with different (and accessible) ways to (re)present data [see for example: Stride's (2014) use of critical, creative non-fictional stories; Fitzgerald's (2009) use of posters; Hooper et al.'s (2021) and Sharpe, Coates and Mason (2021) use of cartoons; and Pang's use of radio plays^5^ Whilst we are optimistic about the future of youth voice research within PE, we are conscious that there is a need to give some thought to what it means to utilize this approach in practice.

## The Four Principles of Student Voice Work

Within the context of school-based student voice research we have drawn upon the work of Robinson and Taylor (2007) and their four principles as a reference point: (1) a conception of communication as dialogue; (2) the requirement for participation and democratic inclusion; (3) the recognition that power relations are unequal and problematic; and (4) the possibility for transformation and change. These have offered us an overarching lens for conceptualizing student voice and also guided our subsequent reflective discussions.

### A Conception of Communication as Dialogue

Early on in our discussions regarding dialogue we have noted a tendency within publications, and indeed through exchanges with other colleagues, to use the term “voice” in a way that implies a verbal exchange. Through our research we have come to recognize the limitations of appropriating voice in this rather limited way. Indeed, according to Robinson and Taylor (2007), creating dialogue should move beyond speaking as the only effective means of engaging with a range of young people. Rather, the term “voice” must be used in inclusive ways and encompass how young people variously articulate their opinions and insights, including through the use of silence (MacLure, 2009; Mazzei, 2009; Finneran et al., 2021). Creating opportunities for different kinds of dialogue, encapsulating verbal and non-verbal exchanges, is something we have attempted to support within our work. For example, dialogue in Ruth's research was encouraged through a diversity of verbal and non-verbal means as she was wishing to promote engagement amongst a range of girls—those experiencing learning difficulties, those with physical disabilities, and those for whom English was their second language. Focus groups were utilized to not only facilitate student-led conversations but for the girls to share their thoughts and ideas about PE through a personal research book and the creation of a series of research artifacts. These were sufficiently varied in Ruth's attempts to consider different girls' communication preferences and needs and included drawings, stories, collages, and mind maps. This enabled the girls to relay information in different ways, not limited to an adult-centric verbal exchange (Robinson and Taylor, 2007).

Dialogue can also be considered in terms of who researchers have dialogue with. On this issue, the youth voice project has been criticized for not acknowledging the perspectives of different young people (Hadfield and Haw, 2001; Burke et al., 2017). The tendency to seek an homogenized voice has been troubled by Robinson and Taylor (2007, p. 6) who claim “such a monolingual assumption is illusory”. Rather, young people should be accounted for and acknowledged as being at the intersections of various identity categories such as age, class, race, ethnicity, gender, sexuality and disability. These collectively contribute to the diversity of young people's lived experiences (Rudduck, 2006). In Annette's study, whilst the majority of the young women involved were “South Asian” and “Muslim”, these labels could not be read as indicators of homogeneity. For example, many factors contribute to the diversity found within “South Asian” and “Muslim” communities—the significance of history, including country and region of origin, the length of time an individual and/or family have resided in the UK, and links to their home country, alongside different cultures, degrees of religiosity, and differing interpretations of Islam (Shain, 2003; Benn et al., 2011). For the girls in Annette's study, issues of class, gender, and ability added to this complexity and contributed to their diverse experiences. In highlighting each girl's unique position, Annette's study demonstrated the similarities that these girls shared with some of their White peers, as well as their differences with White girls and with each other. In so doing, this study goes some way in challenging some of the myths surrounding South Asian, Muslim girls' physical inactivity and disinterest in PE. Relatedly, youth voice research that does not recognize differences within groups that are typically viewed in homogenous ways, can contribute to the perpetuation of these kinds of stereotypes.

We have also come to recognize that research that does not acknowledge and engage with a diversity of young people remains complicit in the silencing of some voices, whilst actively promoting and valuing particular kinds of knowledge. With this in mind, Hadfield and Haw (2001, p. 494) note a tendency for research to “fall back on those who are verbally articulate and self-confident”. Jackson and Mazzei (2009, p. 48) adds,

We seek the familiar voice that does not cause trouble and that is easily translatable. We seek a voice that maps onto our ways of knowing, understanding, and interpreting. A more productive practice, however, would be to seek the voice that escapes our easy classification and that does not make easy sense—the voice in the crack.

We agree with Bragg (2001, p. 70) and Mazzei (2009) who argues that the exclusion of some voices “points to the existence of an ‘implicit contract' to ‘speak responsibly, intelligibly and usefully”', a contract that tacitly seeks those voices that most closely align to organizational cultures and philosophies. Bragg (2001, p. 70) advocates for the inclusion of those voices that are “incomprehensible, recalcitrant or even obnoxious” to ensure youth voice research is reflective of all the young people it claims to represent. In much youth voice research an “implicit contract” to speak intelligibly precludes many young people. These discussions are pertinent to Robinson and Taylor's (2007) second principle which focuses on democratic inclusivity and notions of participation.

### The Requirement for Participation and Democratic Inclusivity

This second principle has encouraged us to consider who we have worked with, how these young people were selected, and the ways we have worked with them. In Robinson and Taylor's (2007) terms, democratic inclusivity highlights the need for a diversity of young people to be involved in research projects. We are all confident that in working with young disabled students, and girls from minority ethnic communities, our research has engaged with different kinds of young people who traditionally have been marginalized in PE research and practice. However, we are mindful that this has not automatically equated to democratic inclusivity. Here we have questioned how diverse our participants were in relation to their school population. And indeed, whether this diversity moved beyond Bragg's (2001) “implicit contract” of youth voice research mentioned earlier. Our discussions have led us to consider how the young people involved in our projects were chosen and what wider processes informed this.

Regarding wider processes, we are all acutely aware that the school context influenced which young people were involved in our research, and ultimately became “the voice” for their peers. For example, Annette's study was initiated with democratic inclusivity in mind as all girls in 1 year group were offered the opportunity to participate. With over 90 students expressing an interest, a number of criteria were put in place to reduce the numbers and make the project manageable. The final 23 girls were chosen based on their ability to return consent and assent forms, and to get themselves organized into small focus groups. The school was also keen to ensure only girls who were “progressing well” in their academic studies were involved. This school directive limited the possibilities of democratic inclusivity particularly in relation to those students not deemed to be sufficiently progressing against markers of academic attainment. Annette had to reconcile that her aspirations for democratic inclusivity had to be compromised in order to secure access to the girls at the school. Similarly, whilst Ruth adopted an inter-categorical methodological approach (McCall, 2005) to ensure girls from multiple social groups were included in her study, she still found herself constrained by the school's dictate. Ruth found herself working with teachers to identify students who may benefit from, and be a benefit to, the project. Upon reflection, this ensured that those students considered more challenging to work with may have been excluded.

In addition to our discussions regarding how young people were chosen for our studies, we also recognize it is important to consider how we are working with these young people. Like Robinson and Taylor (2007), we believe making young people feel comfortable in expressing their thoughts is critical to supporting an inclusive youth voice environment. On this issue, Rudduck (2006) and Jones and Bubb (2020) note that many young people involved in youth voice work are often uncomfortable with providing criticism, anxious of reprisals, and feel pressure to articulate what they think adult stakeholders want to hear. Because of the hierarchical nature of schools, and position of young people as learners, we have each tried to delineate between school activities and the distinctive youth voice projects we were facilitating. Even if only temporary, we saw this as a symbolic and material departure from school enforced rules and hoped this would be more conducive to our youth voice work. For example, for youth voice sessions that took place in lesson time, Annette and Ruth took no issue with girls bringing snacks and drinks, using their mobile phones and playing music—all practices that were against school rules. Ruth also presented each girl with her own personal research book, explaining that they could decorate, draw on, and graffiti this in any way they wanted to make the books more personal to them. This opportunity ran counter to school practice that would have deemed the girls' artwork as defacing and disrespectful. Ruth's account from her research diary highlights her surprise at how beneficial these small concessions were to fostering open conversations:

I'm a little shell-shocked but some of them really opened up. At times I forgot that I was a researcher. I just sat and listened, mesmerized, getting lost in their words. (Ruth's Research Diary)

Despite our initial beliefs that the spaces we were creating were supportive and inclusive, this was not always the case. As Annette reflects, at the end of the last focus group session two White girls (the ethnic minority in this school and the research) stayed behind and began to share issues they had never raised before. Discussions about their White identity, feeling different, their difficulties in being accepted, and how this influenced their experiences of PE and their education more broadly were expressed. This exchange left Annette with a number of questions. She queried the inclusive nature of the project, and speculated on how she could have better considered these girls' additional needs, perhaps by providing alternative and non-collective ways for them to share their stories? It was a stark reminder for Annette, that whilst she believed she had created an inclusive environment, this may not be what her research participants experienced. This encounter highlighted how each of the girls in the study were differently positioned within broader socio-cultural and material relations which influenced their ability to express themselves (James, 2007; Jackson and Mazzei, 2009). The experience also signaled to Annette the importance of silence, and the inadequacies of voice (MacLure, 2009). Jackson and Mazzei (2009) work is useful here in encouraging researchers to not just attend to *what* we hear, but *how* we hear. She argues that “we must listen for, listen to, and ask questions not just in response to an answer voiced, but to ask questions of a withheld response, a non-response, or a masked response” (p. 54).

For Ruth, her immediate concerns around an inclusive environment focused on the needs of students with disabilities and those who spoke English as a Second Language (ESL). Ruth considered these in her planning of the focus group sessions. For example, when offering the students written materials she ensured they were printed out in large font to aid those with a visual impairment or specific learning needs. She consulted with the ESL teacher to gain a better understanding of the students' language capabilities to inform the ways in which information was conveyed. These strategies were used consistently for all participants so that no individual student was singled out. However, another element of providing a safe, inclusive space, one which was less appreciated, was student relationships. Midway through the research, issues of bullying for a number of Slovakian girls became apparent. Upon reflection, encouraging them to discuss personal experiences in the presence of other girls may have exacerbated the problematic relationships they had with some of their peers. In retrospect, the use of checklists and feedback forms devised from Lundy's (2007) Model of Participation by the Irish Government's Department of Children, Equality, Disability, Inclusion, and Youth^6^ might have helped us to better understand how inclusive our research spaces actually were. In relying on our adult centric assumptions it is difficult for us to argue with confidence that the environment we created was as conducive to open, safe and supported conversations as first thought. Questions remain regarding how comfortable these young people felt in expressing some of their most intimate thoughts and feelings, whether all students engaged as fully as they would have liked, or whether we had a tendency to focus on those who were more willing to share with us.

Robinson and Taylor's (2007) second principle is also concerned with how to promote youth voice, and we have taken this to include working in participatory ways. Whilst there are many advocates of participatory research, Gallacher and Gallagher (2008) note that much work claiming to be participatory is simply an extension of more traditional researcher controlled methods. In contrast, Dentith et al. (2012) believe participatory research should move beyond methods and embrace all stages of a study—conceptualizing, designing and conducting the research and interpreting, reflecting and acting upon the data to implement change. As such, Enright and O'Sullivan (2010) advocate for the positioning of researchers as “adult allies” who enable and facilitate young people's participation throughout the research process. On reflection, we feel that we have drawn upon these understandings to differing degrees in our various studies. For example, during her PhD Ruth asked the students to set the parameters of the project by deciding what aspects of PE would be the focus. From these discussions she designed observation templates that she could use during PE lessons. A consistent insight emerging from the focus groups was the frustrations students experienced in not understanding what the teacher required them to do. Based on this, Ruth's observation template included the communication styles of the teacher. Thus, the observations were guided by the voices of the students, rather than being led solely by the ideas of the adult researcher. However, despite Ruth's efforts to consider participation, this was not without some challenges. For instance, although the students offered their ideas regarding which PE areas were pertinent to focus upon, the broad topic of PE was already determined. As the recipient of a PhD bursary, the boundaries of Ruth's study were clearly defined and thus the research agenda of experiences in PE was non-negotiable.

For Annette, her initial (and with hindsight, somewhat naive) understanding of participation focused on the methods employed in her study. Noting the importance of fun (Fox, 2013), she became preoccupied with making her data collection phase interactive, engaging and enjoyable for the girls. Looking back there are two limitations to her thinking around participation. First, methods in their own right do not make for participatory research. Second, Annette's ideas around the kinds of activities that might engender fun, enjoyment and interaction might not be viewed in quite the same way by the girls. With Annette's data collection phase taking place in a school, and indeed in a lesson, she has since come to realize that the tasks she created may not have been perceived by the girls as an entertaining alternative to schoolwork but rather an extension of the many tasks given as part of the typical school day. Relatedly, the ways they girls often viewed Annette, as a teacher, would have undoubtedly influenced their “participation” in the activities she facilitated. The relationship between the researcher and those being researched and how this influences the research process, including the kinds of data being generated and opportunities for student voice to be captured is considered with reference to Robinson and Taylor's (2007) third principle.

### The Recognition That Power Relations Are Unequal and Problematic

This third principle has encouraged us to explore power relations and consider how power might be re-balanced in our youth voice projects (Robinson and Taylor, 2007). Like others (Nelson, 2017; Charteris and Smardon, 2019; Finneran et al., 2021), we are skeptical of claims that youth voice projects can disrupt traditional power relations within research. Power operates within the research context and through the research process, and this needs to be recognized in any youth voice project. For us, this should include considering the relations between different individuals. That is: relations between adult researchers and their research participants; the adult researchers and other adult stakeholders; other adult stakeholders and the research participants; and the relationships between the young people themselves. Hadfield and Haw (2001, p. 494) argue that young people can be “particularly susceptible to certain forms of manipulation because of the power relationships in which they are caught up”. As researchers working with young people in schools, we are particularly aware that our research contexts are not impermeable vacuums. Rather they are influenced by the pervasive power hierarchies structuring schools and society more broadly, and the wider socio-cultural material conditions of our lives. These, in turn, inform the ways we, as researchers and research participants, engage with each other, and the discussions that take place. This is something we have grappled with in terms of reconciling the extent to which power dynamics can be re-balanced in any way.

From the outset of our school-based projects we were conscious that as adults we would likely be viewed as teachers. Because of the ethos underpinning our projects we were reluctant to be positioned in this way and made concerted efforts, similar to O'Brien (2019), to distance ourselves from this identity. Both Annette and Ruth undertook a series of conscious and continuous acts of resistance to “mute power” associated with the role of teacher (Fitzpatrick and Allen, 2017). For example, Annette physically positioned herself away from the PE teachers during her observation of lessons, chatted to girls informally, and did not display her school staff card. When girls called her “Miss” she politely reminded them that they could call her Annette. And, when girls apologized for not bringing research artifacts to focus groups, she reminded them it was their choice to bring or complete them. Similarly, Ruth consciously used her first name and attempted to use a friendly, conversational style when interacting with students to position herself differently to the teachers. Moreover, she consciously chose not to make notes during lessons, to avoid any feelings that the students were under observation from an authority figure.

Often these strategies did not mute our teacher power and identity that the young people had appropriated for us. On a number of occasions, our teacher identity was reinforced rather than diluted. Interestingly, it was not just the young people who were positioning us as teachers. This is evident in both Annette's and Ruth's diaries:

In today's lesson a teacher came into the dance studio with a pupil and told me she had caught her truanting. Despite my assertions that I was a student observing the class, and pointing out the PE teacher, I was told quite curtly that I had to report it whoever I was. (Annette's Research Diary)[Teacher] turns to me, wringing her hands, clearly quite agitated. “I haven't had any time to plan for this lesson and I hate football! You're a football coach, right? Can you help?”. I'm flattered but also a bit wary. Obviously, I'm more than happy to help her, so I lead a simple session. I'm delighted that she seeks my help and advice. However, I don't want the students to see me as “a teacher”. I want them to see Ruth. I'm careful throughout the lesson to tiptoe that fine line. (Ruth's Research Diary)

These episodes crystallize how our identity was read as teacher by those around us rather than a researcher (O'Brien, 2019). We came to (reluctantly) accept that the young people we were working with (and others in the school) would make their own decisions regarding who and what we were.

Another way we attempted to challenge power relations was through our communications with students. Robinson and Taylor (2007) argue that from the outset those wishing to undertake student voice work must recognize that power influences all processes of communication. From the beginning of our studies we were cognisant that being White, non-disabled adults with no former connections to the schools would create a number of communication barriers with these young people. We each tried to counterbalance this in a number of ways, for example, by spending significant periods of time in the schools observing practice prior to approaching the young people about their involvement in our projects. To some extent, doing this enabled us to get to know the young people, and them us, as well as increasing interest in our youth voice research:

On reflection I think the observations allowed me to build up a rapport … one that I think has proved useful in recruiting participants. Certainly last Friday, when I visited a group I have not seen since July, I was greeted with waves and hellos which made me feel welcome. Girls I have never spoken to are stopping me in the corridor to ask me if they can be involved and if I will come to their form. Girls are approaching me in the changing rooms to ask to put their names down. (Annette's Research Diary)It's been nine months since I started this research journey and set foot in the school … It's really heartening to reflect on how my relationships with students have grown. There's little things, like the high-fives I get in lessons, or them coming over to talk to me in the corridors. I missed last week's PE because I was ill, and [students] came to ask me where I was. That makes me feel like a proper part of their school lives. (Ruth's Research Diary)

Building on these developing relationships we each also wanted our communication with the young people to be more of a mutual and collective endeavor (Robinson and Taylor, 2007; Flintoff et al., 2008). To work toward this aspiration we utilized more interactive, participatory approaches to working with the young people and generating data. In part we anticipated these alternative approaches would trouble the power dynamics often evident in traditional interviews. We also believed using a variety of participatory methods would appeal to different learning styles, needs, and preferences. As such, using these approaches would enable a diversity of students to participate (whether through drawing, writing or verbal exchanges) in ways that were inclusive, accessible and would place the students as the expert in their lives (Hadfield and Haw, 2001). For example, in Annette's study the girls presented the various research artifacts they had chosen to create. Some discussed the important people in their lives they had captured through photos. Other girls focused upon places of meaning to them and highlighted this through mapping exercises, and some chose to share their likes and dislikes in PE by creatively decorating cardboard boxes. These collective ways of communicating, and generating data with the young people, also opened up opportunities for them to share and reflect more widely on their experiences. In particular, Annette noted how some of the girls were becoming more confident in articulating their views. Some of these discussions focused on the inequities they observed at school and in their broader lives. For example: they began to question why they couldn't play cricket and football in PE like the boys; why they couldn't go boxing in the evenings like their brothers; and why certain community members disapproved of them engaging in physical activity in the streets. Annette viewed these ephemeral moments as important junctures in the girls developing their critical consciousness. Here, they began to challenge some of the taken for granted practices that operate through their schools, families and communities and that have repercussions on their daily lived social realities. Despite Annette's delight that these girls were beginning to think critically she was less optimistic that this would lead to any longer term change within the school. As Hadfield and Haw (2001, p. 496) warn, “for all the effort and time put into the numerous projects that have tried to get the ‘voices' of young people heard, their widespread impact has been called into question” resulting in little influence on policy or practice. The possibility for transformation and change is the final principle of Robinson and Taylor's (2007) student voice work and we discuss this next.

### The Possibility for Change and Transformation

In their final principle, Robinson and Taylor (2007) suggest that listening alone is not sufficient in student voice work. In their view the insights young people share should be acted upon. Similarly, Hadfield and Haw (2001, p. 496) note that:

Although there are numerous personal benefits claimed for young people involved in “voicing” their views and experiences this is not generally their sole motivation. Generally, young people want things to change, something to happen, somebody to take note. They want to have an impact.

Robinson and Taylor (2007) identify a continuum of student voice work in relation to transformation. At one end of the continuum is tokenistic work, superficial listening with an acknowledgment that consultation has taken place. Pearce and Wood (2019) believe there are a number of reasons for tokenistic engagement in schools: not having authentic intentions; external pressures to teach in ways approved by Ofsted^7^; and students being schooled into adopting passive roles. Relatedly, Robinson and Taylor (2007) note that school councils can be tokenistic because they tend to attract students who are confident, less suspicious of authority, and also often personally benefit from the school systems underpinning this. They conclude that these forums “listen to only the articulate and able … and to those who agree with what the school wants to hear” (p. 10). At the other end of the continuum are those projects that engage young people in ways that lead to changes in school policy and practice. This positioning is also reflective of Hadfield and Haw's (2001) understandings of voice work with young people. Here, there is a move away from merely expressing a point of view to a more involved act of participation. This latter approach encourages engagement between people and organizations to help better shape the lives of the communities they serve. Like Quennerstedt (2019), we recognize that approaches that encourage freedom of expression, creativity and individuality are increasingly important when young people's views, needs and perspectives are ignored in favor of adults' voices and reasoning. But, we are also cognisant that such projects are impacted by wider politics and priorities evident in schools. According to Pearce and Wood (2019), the neoliberal and performative climate pervading education is hampering projects promoting student voice. On this issue Jones and Bubb (2020) believe there is a tension between student voice research and broader school improvement agendas. The former is more likely to be driven by a democratic and bottom-up approach, whilst school improvement is often underpinned by top-down agendas led by measures associated with efficiency and excellence.

On an individual level, we have seen first hand how young people involved in our projects can respond positively to opportunities to share and discuss various issues. Indeed, as Annette highlighted earlier, in a very tangible way this led to expressive episodes that stimulated some critical awareness. Moreover, toward the end of Annette's project the girls were able to share how they felt the project had helped them more broadly:

“I am more aware of myself”, “I could express myself”, and “more confidence to talk more.” (Annette's Research Diary)

We are heartened by these possibilities but also mindful that these moments of criticality do not necessarily translate into any significant, longer term transformation. As Hill Collins (2000) notes, this requires some form of collective action. With regards to this collective action, Ruth initially planned to work with teachers and students to enable curriculum change. Inspired by the action research projects of Oliver et al. (2009) and Enright and O'Sullivan (2010), her vision was to support students to co-create a PE curriculum. However, at the time of her research the school was due an inspection from Ofsted. With this imminent inspection Ruth was not offered access to the teachers and consequently the focus of the project changed. As such, Ruth's ambition to transform the lives of the students and the practices of the PE department was not possible. At the time of this change Ruth was frustrated and felt her ambitions to promote transformation had failed to materialize.

This has been such a frustrating few weeks. The focus groups are going great. The girls seem to be opening up, and pointing out areas that could easily be improved to better their experiences. But, because of something completely out of our control, it seems like it will go nowhere. Teachers no longer have the time to meet with me and the students to try and understand. I feel really bad for the students. I feel like I've let them down in some way, like I've led them on. (Ruth's Research Diary)

Whilst we have attempted to re-balance power in order to seek transformation, we are aware that this will always be limited by broader power structures that influence the lived experiences of young people in schools. This has perhaps made us appreciate and value even more those momentary signs of individual or collective expression that point to a realization of injustice or inequality. Whilst these may have been nothing more than passing remarks, we agree with Cook-Sather (2002) that you should not underestimate these discursive gestures as they may become the segue needed to open up the possibilities for transformation and change.

As we have already indicated, the connectivity we had within our schools with those positioned to reframe PE was not how we had initially envisaged. There were occasions though where we did manage to make some positive inroads. For Annette, the possibilities of change seemed more likely through the unplanned, informal conversations she had with staff.

The Head of Dept asked me my opinion on what to do with girls in rounders who do little activity (e.g., may hit the ball once or go deep field and rarely touch the ball). I spent some time explaining that for some girls this is a good way to hide because they don't like PE. I went into more detail about why girls like to play with friends—not because they like to mess about, but because they feel more confident about having a go and not getting laughed at. We seemed to agree that smaller games would work more. Also got the opportunity to talk about girls' dislike of hockey and desire to play football and cricket. He seemed amenable to the idea of 5 a side football. (Annette's Research Diary)

On reflection, we now recognize these conversations with teachers were in fact a critical means of connecting the students' perspectives to staff in the schools. Of course, we were also aware that we were only able to reach out to those teachers who were willing to start a conversation with us. For Ruth, one teacher in particular was receptive to the suggestions she made. This discussion emerged after the girls involved in her research shared their frustrations with the PE teacher's attitude toward them. For one student, experiencing a visual impairment, the teacher's reliance on visual forms of communication was challenging. As Ruth reflects, drawing the teacher's attention to this increased her awareness and she subsequently adapted her approach to communicating with the student.

It's been really heartening to see [student] within PE over the last few weeks. [teacher] really seems to have taken the information on board. It's only small changes, like printing the learning objectives out in much bigger font, or even reading them out loud. Or during the lesson, being much more mindful of her demonstrations and where [student] is sitting, or even checking in with [student] to ensure she understood. But seemingly small changes like this seem to have made a world of difference for [student]. It's great that [teacher] has been so receptive to this information, and hasn't taken it as a critique. (Ruth's Research Diary)

Similarly, a teacher at Annette's school took the time to read some of the narratives that were crafted. The teacher disclosed that these had moved her emotionally and this had prompted her to begin to change some of her practice, including introducing new activities which the girls had suggested.

I found myself with an opportunity to share some of the girls' critical non-fictional narratives with [teacher]. She took her time reading them and from her reaction I could see she was moved by some, whilst finding others entertaining—I am pleased as this is what I was hoping for—to connect the girls' experiences to those reading about them. On finishing, [teacher] shared how upset some of them had made her feel particularly when it was clear how PE was negatively impacting upon them. She also shared her frustrations around why some of the activities remained on the curriculum despite girls' clear dislike of them. (Annette's Research Diary)

It is clear that there have been some modest possibilities for change of the teachers' practices and girls' thinking. These are essentially small incremental changes and as such do not signal any kind of cultural shift. Such a shift would require a wholehearted desire and commitment of more teachers and at the same time a recognition that the girls are well placed to offer insights into their PE experiences.

## Concluding Remarks

This paper offers some reflections of our attempts to promote school-based student voice research. Through this research we have come to recognize that our aspirations for student voice research are actually very difficult to achieve. Indeed, our collective reflections have made us more cognisant of the various factors that can impact upon what is possible through student voice projects. These possibilities are couched in pragmatic concerns around time, space, skills and relationships. But there are also broader social structures and cultures which are ever present within school-based research to consider. In combination, these pragmatic and structural impositions of power can serve to limit rather than enable student voice aspirations. In particular, student voice research in schools may curtail rather than be enabling of understandings of the daily lives and experiences of young people (Fitzgerald et al., 2020). Nor will this approach always support transformative changes. Through our collective reflections we now wonder if we were misguided and somewhat naive about the assumptions we made about the possibilities that student voice research brings. Although now, we feel in a position where we have reconciled the (im)possibilities that student voice research brings. To end this paper, we would like to offer a number of concluding remarks.

First, in our student voice research we have claimed the authoritative position by initiating the research and also making the assumption that young people actually need our help to bring to the fore their views about PE. This outlook seems counter to the ethos guiding student voice work. In our case, as adult researchers we had fallen into a paternalistic trap of presuming young people need our support to articulate their views or enact change. Whilst we have supported alternative ways of listening and sharing with young people in our research activities, we have not sufficiently acknowledged the different ways that young people mobilize and resist PE practices. For example, non-participation in PE, forgetting kit, and taking certain positions in team games, are conscious reactions to PE and these are important signals of how young people view and experience PE. Young people do have a voice and can act upon this, but within a school system this is not necessarily recognized by researchers, teachers and other staff. Relatedly, we are mindful that the neo-liberal culture permeating schools is likely to curtail rather than be an asset to student voice endeavors. It is clear to us that it is not young people who need help to share their insight and experiences, it is in fact us—researchers, teachers and schools—who need help to better understand how we can more readily recognize and be attentive to young people's voices.

Second, we have increasingly come to recognize that the possibilities for change and transformation through student voice research may be more modest than we had envisaged. For us this was evident through the young people's heightened critical awareness and also discussions with teachers that led to changes in specific aspects of their practice. Whilst modest, these inroads are critical, and we believe should not be dismissed as insignificant. Collectively, they are important starting points for supporting more sustained efforts to bring into question dominant power relations circulating in schools. Beyond the immediacy of any student voice projects, what we are pointing to here is for this kind of work to become core rather than considered an addendum to the activities in schools. As well as managing our own expectations of the possibilities for change, we have also become aware of the need to more thoroughly consider how we can better support the expectations of the young people involved in student voice research. We see this as a delicate undertaking. For instance, managing expectations around what is achievable, what are realistic outcomes, what should be prioritized, and where compromises might be needed. As Hadfield and Haw (2001, p. 495) suggest, there is also benefit in young people becoming knowledgeable about their audiences as this enables them to be “in a position to strike a better balance between being listened to and challenging professionals sufficiently to change their practice”. Of course, supporting this kind of dialogue can also be read as an assertion of authority in itself. That is, as adult researchers we are appropriating what benefit should be derived and directing young people in a particular way. Implicitly this reinforces power differentials within student voice work.

Third, in undertaking our student voice research we recognize, like Enright and O'Sullivan (2010), the need to secure adult allies to support activities and potential outcomes. We have been able to broker gatekeeper allies which has then enabled access to schools and young people. However, we have been less successful in securing sustained allies during our student voice projects. On reflection we have perhaps not sufficiently attended to ally building beyond that needed to secure access to schools. Once this was secured our preoccupations centered on the delivery of the student voice projects with the students. In hindsight we should have paid more attention to ally building and also considered how specific allies in decision making roles or with access to resources could have been more closely aligned to our projects. Mobilizing these allies, as agents of change, may have enabled actions to be initiated based on the students' insights and desires for PE. Of course, gaining support from a collection of allies also requires careful negotiation and a sensitivity to ensure the voices of young people and their ideas remain at the heart of any discussions and subsequent change. Caution is needed here, as we are mindful that some may represent themselves as allies but are merely appropriating a student voice project for their own ends. Critically, as researchers we should not underestimate the possibilities of us as culpable allies as well.

As we have discussed elsewhere, engaging in student voice research is an immersive and messy encounter that involves navigating a journey that is anything but straightforward (Fitzgerald et al., 2020). Even though this is the case, our moral and ethical compass continues to point us in this direction, and we remain firm advocates of student voice work.

This is not because we feel that work of this kind will lead to more authentic understandings that can be acted upon to resolve some complex societal issues affecting young people. As Jackson and Mazzei (2009, p. 47) argues, the “promise of a voice that can provide truth, fixity, knowledge, and authenticity” should be abandoned. Like any research participant's account, those provided by young people are partial, changeable, contradictory, and must be considered within the structural and cultural conditions within which they are formed. As James (2007, p.265) articulates, the voices of our participants must “be acknowledged in their particularity and the generalizations we draw from them must continue to be carefully crafted. Indeed, they must be recognized as crafted; their ‘authenticity' must be interrogated, not assumed”. With this in mind, our moral and ethical compass for undertaking student voice research is guided by our belief that young people should be consulted on issues that are important to them and affect their lives. As such, we continue to grapple with the merits and challenges to utilizing student voice research and continue to reflect upon the possibilities this kind of approach brings.

## Data Availability Statement

The original contributions presented in the study are included in the article/supplementary material, further inquiries can be directed to the corresponding author.

## Ethics Statement

The studies involving human participants were reviewed and approved by Leeds Beckett School of Sport. Written informed consent to participate in this study was provided by the participants' legal guardian/next of kin.

## Author Contributions

All authors listed have made a substantial, direct, and intellectual contribution to the work and approved it for publication.

## Conflict of Interest

The authors declare that the research was conducted in the absence of any commercial or financial relationships that could be construed as a potential conflict of interest.

## Publisher's Note

All claims expressed in this article are solely those of the authors and do not necessarily represent those of their affiliated organizations, or those of the publisher, the editors and the reviewers. Any product that may be evaluated in this article, or claim that may be made by its manufacturer, is not guaranteed or endorsed by the publisher.

## Footnotes

^1^Throughout this paper, we use the terms “youth voice” and “student voice”. While we understand youth voice to be the broad practice of attempting to work *with* young people in research, and which can take place in a variety of contexts, we use the term student voice to refer to this kind of research activity in schools—in our case working in secondary schools.

^2^We use the term critical, creative non-fictional narratives to acknowledge that the narratives are crafted from the data generated by our research participants. They are critical in their ability to stimulate discussion, and creative through the use of fictional techniques. For more information on the use of alternative means of re-presenting data we recommend Sparkes (2002) text, Telling Tales in Sport and Physical Activity, particularly the chapter on Fictional Representations.

^3^For more information on The Education Act 2002 see www.legislation.gov.uk/ukpga/2002/32/contents.

^4^For more information on The Children Act 2004 see www.legislation.gov.uk/ukpga/2004/31/contents.

^5^https://www.chapelfm.co.uk/elfm-player/shows/list/radioplays-on-british-chinese-youth/.

^6^https://hubnanog.ie/participation-framework/.

^7^Ofsted is the “Office for Standards in Education”, who inspect and regulate teaching and learning standards within schools in England.
